# Intraspecific divergence in essential oil content, composition and genes expression patterns of monoterpene synthesis in *Origanum vulgare *subsp. *vulgare *and subsp. *gracile *under salinity stress

**DOI:** 10.1186/s12870-023-04387-5

**Published:** 2023-08-07

**Authors:** Zahra Azimzadeh, Abbas Hassani, Babak Abdollahi Mandoulakani, Ebrahim Sepehr, Mohammad Reza Morshedloo

**Affiliations:** 1 Department of Horticultural Science, Faculty of Agriculture, Urmia University, Urmia, Iran; 2 Department of Plant Production and Genetics, Faculty of Agriculture, Urmia University, Urmia, Iran; 3 Department of Soil Science, Faculty of Agriculture, Urmia University, Urmia, Iran; 4 Department of Horticultural Science, Faculty of Agriculture, University of Maragheh, Maragheh, Iran

**Keywords:** *Origanum vulgare* L., Salinity stress, Gene expression, 1-deoxy-D-xylulose-5-phosphate reductoisomerase, γ-Terpinene synthase, Carvacrol

## Abstract

**Background:**

Oregano (*Origanum vulgare* L.), one of the important medicinal plants in the world, has valuable pharmacological compounds with antimicrobial, antiviral, antioxidant, anti-inflammatory, antispasmodic, antiurolithic, antiproliferative and neuroprotective activities. Phenolic monoterpenes such as thymol and carvacrol with many medical importance are found in Oregano essential oil. The biosynthesis of these compounds is carried out through the methyl erythritol-4 phosphate (MEP) pathway. Environmental stresses such as salinity might improve the secondary metabolites in medicinal plants. The influence of salinity stress (0 (control), 25, 50 and 100 mM NaCl) on the essential oil content, composition and expression of 1-deoxy-D-xylulose-5-phosphate reductoisomerase (*DXR*), γ-terpinene synthase (*Ovtps2*) and cytochrome P450 monooxygenases (*CYP71D180*) genes involved in thymol and carvacrol biosynthesis, was investigated in two oregano subspecies (*vulgare* and *gracile*).

**Results:**

Essential oil content was increased at low NaCl concentration (25 mM) compared with non-stress conditions, whereas it was decreased as salinity stress intensified (50 and 100 mM). Essential oil content was significantly higher in subsp. *gracile* than subsp. *vulgare*. The highest (0.20 mL pot^−1^) and lowest (0.06 mL pot^−1^) amount of essential oil yield was obtained in subsp. *gracile* at 25 and 100 mM NaCl, respectively. The content of carvacrol, as the main component of essential oil, decreased with increasing salinity level in subsp. *gracile,* but increased in subsp. *vulgare*. The highest expression of *DXR*, *Ovtps2* and *CYP71D180* genes was observed at 50 mM NaCl in subsp. *vulgare*. While, in subsp. *gracile*, the expression of the mentioned genes decreased with increasing salinity levels. A positive correlation was obtained between the expression of *DXR*, *Ovtps2* and *CYP71D180* genes with carvacrol content in both subspecies. On the other hand, a negative correlation was found between the expression of *CYP71D180* and carvacrol content in subsp. *gracile*.

**Conclusions:**

The findings of this study demonstrated that both oregano subspecies can tolerate NaCl salinity up to 50 mM without significant reduction in essential oil yield. Also, moderate salinity stress (50 mM NaCl) in subsp. *vulgare* might increase the carvacrol content partly via increment the expression levels of *DXR*, *Ovtps2* and *CYP71D180* genes.

## Background

**Fig. 1:**
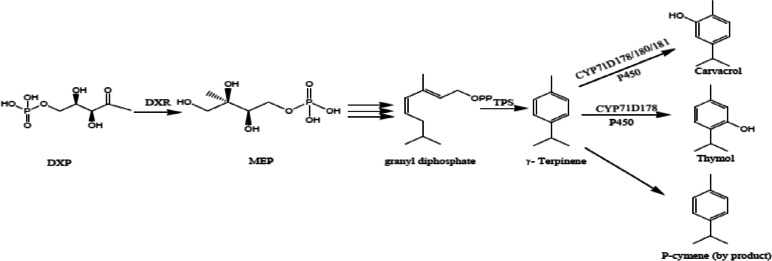
Proposed pathway for carvacrol and thymol biosynthesis in oregano and thyme in plastids (Crocoll, 2011). *DXR*: 1-deoxy-D-xylulose-5-phosphate reductoisomerase, *Ovtps2*: γ-Terpinene synthase, *CYP71D180*

Oregano (*Origanum vulgare* L.) is an herbaceous perennial plant in the mint family (Lamiaceae), native to Europe and central Asia [1, 2]. Many investigations have demonstrated its antimicrobial, antiviral, antioxidant, anti-inflammatory, antispasmodic, antiurolithic, antiproliferative, and neuroprotective pharmacological activities [3–5]. Furthermore, it is an important natural source for preserving foods or cosmetics due to its high antioxidant activity [5–7]. A great variety has been reported in the essential oil composition of oregano, which is attributed to the high morphological and chemical diversity within the genus *Origanum* [8–10]. Depending on the growth conditions, growth stage and different organs, the dominant constituents of essential oil in oregano have been recognized such as carvacrol, thymol, *p*-cymene, γ-terpinene, sabinene, linalool, borneol, β-bisabolene, terpinen-4-ol, β-caryophyllene, caryophyllene oxide, germacrene D and β-ocimene [5, 9, 11–15]. Phenolic monoterpenes (thymol and carvacrol) identified in *O. vulgare* essential oil, are medically important due to their antioxidant, antimicrobial, antitussive, expectorant, antispasmodic and antibacterial properties [16–19]. Terpene synthases (TPS) are key enzymes involved in the biosynthesis of monoterpenes and sesquiterpenes, which catalyze the oxidation steps from precursors for each group of terpenes [20, 21]. Terpene synthase genes have been identified in different species of mint family plants such as, *Salvia officinalis* [22, 23], *Mentha spicata* [24], *O. vulgare* [25], and *Thymus capitatus* [26]. The methyl erythritol-4 phosphate (MEP) pathway provides substrate for producing both mono and di-terpenes in plastids [27]. The induction and activity of 1-deoxy-D-xylulose 5-phosphate reductoisomerase (*DXR*) is recognized as an important key point in MEP pathway, which has been reported as the first major and separating step of this pathway [21, 28]. Generally, the synthesis of geranyl diphosphate (GDP) from isopentenyl diphosphate (IPP) and dimethylallyl diphosphate (DMAPP) (as a precursor of monoterpenes) catalyzes by geranyl diphosphate synthase. The next step of the pathway is the conversion of GDP to γ-terpinene, which is catalyzed by γ-terpinene synthase. In plants such as thyme and oregano, the precursor of thymol and carvacrol is γ-terpinene [25, 29] and cytochrome P450 (CYP) monooxygenases are involved in the conversion of γ-terpinene to thymol and carvacrol (Fig. 1). According to the previous studies, carvacrol is derived from γ-terpinene through the acting of *CYP71D180* and *CYP71D181*, whereas *CYP71D178*, *CYP71D179* and *CYP71D182* are likely involved in thymol biosynthesis [30–32].

About 20% of the world’s land, as well as about half of arable irrigated land in the worlds, is affected by salinity [33]. As a worldwide issue, soil salinization restricts agricultural production due to its adverse effect on the plant growth and production [34, 35]. Soil salinity reduces soil water potential, leaf water potential and turgor pressure of the plant cells, consequently induce osmotic stress [36]. High accumulation of ions (Na^+^ and Cl^−^) in saline conditions prevents K^+^ and Ca^+2^ uptakes and leads to ion imbalance [37]. Salinity increases reactive oxygen species (ROS) in the plant cells [38] which causes lipid peroxidation, membrane degradation, and DNA and protein damage [39]. To deal with saline conditions, plants use various strategies such as ionic homeostasis and partitioning, ion transport, osmotic adjustment, antioxidant defense system, and polyamine biosynthesis [40]. Furthermore, plant secondary metabolites notably improve plant growth and survival under biotic and abiotic stresses [41, 42] and their biosynthesis and accumulation are influenced by environmental stresses such as salinity [43]. It has been demonstrated that environmental stresses might change both the quality and quantity of the plant secondary metabolites through influencing the expression of the genes involved in their biosynthesis [44]. Studies have shown that soil salinity changes essential oil biosynthesis and composition in several plant species such as *Salvia officinalis* [45], *Satureja hortensis* [46], and *Melissa officinalis* [47].

To our knowledge, the effect of salinity stress on the content of terpenes and expression of their biosynthetic genes has not been evaluated in *O. vulgare* yet. Due to the presence of valuable compounds in the essential oil of *O. vulgare*, study the expression of the genes involved in their biosynthesis and their association with the accumulation of the compounds under salinity conditions may be of great interest for pharmaceutical and industrial market. Hence, for the first time, the expression of the genes involved in the biosynthesis of the valuable secondary metabolites (carvacrol and thymol) was compared in two oregano subspecies (*gracile* and *vulgare*) under various salinity levels. Moreover, the association between genes expression levels and their corresponded compounds, changes in essential oil content, oil yield and their compounds were also studied under salinity conditions.

## Results

### Essential oil content and yield

**Fig. 2:**
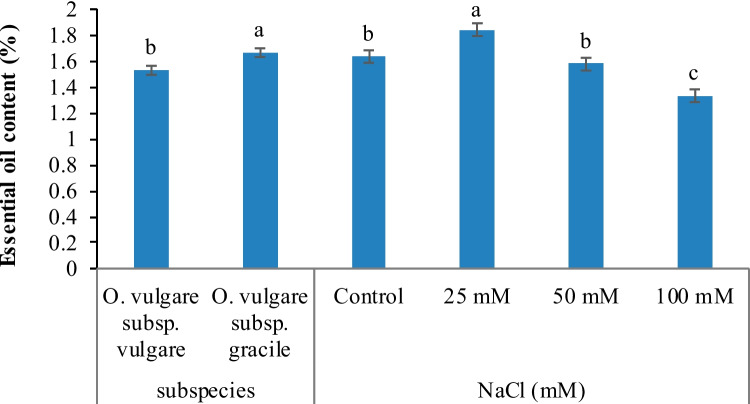
Effect of salinity stress and *Origanum vulgare* subspecies (subsp. *vulgare* and subsp. *gracile*) on essential oil content. Columns with different letters have significant differences (*p* < 0.05)

**Fig. 3:**
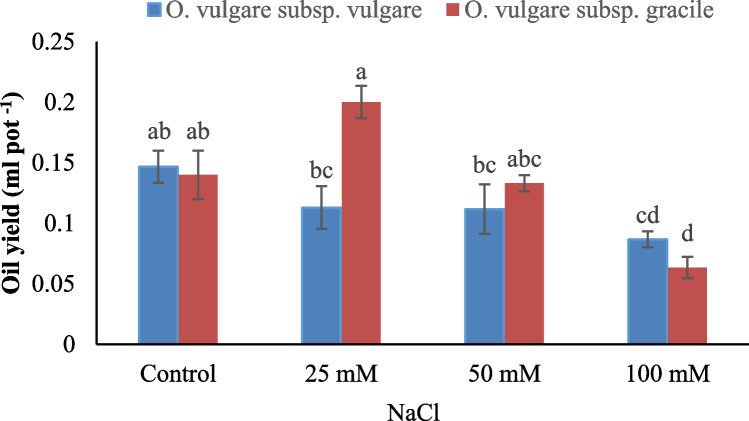
Interaction effect of salinity × *Origanum vulgare* subspecies (subsp. *vulgare* and subsp. *gracile*) on essential oil yield. Columns with different letters have significant differences (*p* < 0.05)

Essential oil content was significantly affected by salinity treatments and subspecies. According to the results, essential oil content was increased at low NaCl concentration (25 mM) compared with non-stress conditions, whereas it was decreased as salinity stress intensified (50 and 100 mM). Briefly, essential oil content was significantly higher in subsp. *gracile* than subsp. *vulgare* (Fig. 2). Essential oil yield was significantly influenced by salinity treatments, subspecies and their interaction. In *vulgare* subspecies, essential oil yield decreased by increasing salinity, but the difference between 0, 25 and 50 mM NaCl was not significant. In *gracile* subspecies, the essential oil yield increased by enhancing the intensity of salinity, up to 25 mM and then decreased by increasing salinity level. Also, the difference between 0, 25 and 50 mM NaCl was not significant (Fig. 3). A positive relationship was found between essential oil content and yield in both subspecies (Fig. 6a,b).

### Chemical composition of essential oil

**Fig. 4:**
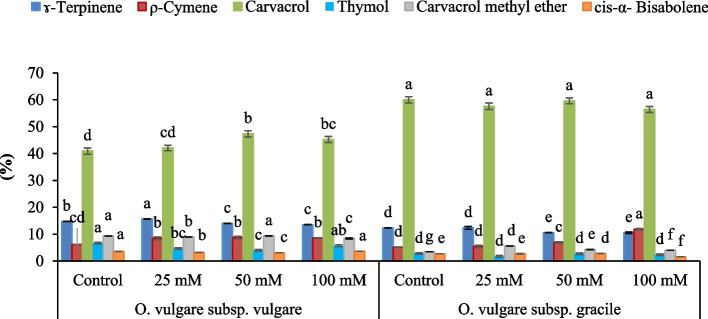
Interaction effect of salinity × *Origanum vulgare* subspecies (subsp. *vulgare* and subsp. *gracile*) on γ-terpinene, *ρ*-cymene, carvacrol, thymol, carvacrol methyl ether and cis-α-bisabolene content in the essential oil. Columns with different letters have significant differences (*p* < 0.05)

The alterations of essential oil compounds in *O. vulgare* subsp. *vulgare* and *O. vulgare* subsp. *gracile* under salinity stress were presented in Table 1. According to the results of GC–MS analysis, total volatile compounds detected in *gracile* and *vulgare* subspecies were 23 and 27, respectively. The dominant constituents of essential oils were carvacrol, carvacrol methyl ether, γ-terpinene, thymol, cis-α-bisabolene and *p*-cymene in both subspecies. The results revealed the different impact of salinity on the chemical composition of essential oil in two subspecies. The highest percentage of carvacrol (60 and 47.36%) was recorded at non-stress conditions and 50 mM NaCl in *gracile* and *vulgare* subspecies, respectively. Although in *gracile* subspecies, the percentage of carvacrol decreased with the application of salinity stress, no significant difference was observed between salinity treatments in terms of this composition. Conversely, in *vulgare* subspecies, the percentage of carvacrol raised by increasing salinity levels. Although, the trend of thymol changes in two subspecies does not follow a discrete pattern, but in both subspecies, the amount of thymol in non-stress treatment was higher than salinity treatments. The findings of this research demonstrated that, *p*-cymene was significantly increased in both subspecies by enhancing salinity stress. However, no significant differences were found between 25, 50 and 100 mM salinity treatments in *vulgare* subspecies. In both subspecies, the amount of *γ*-terpinene increased up to 25 mM NaCl and then decreased by increasing salinity. Furthermore, the trend of changes in carvacrol methyl ether and cis-α-bisabolene did not follow a specific pattern, however, *vulgare* subspecies had higher content of carvacrol methyl ether and cis-α-bisabolene under all salinity treatments (Fig. 4).

**Table 1:** Essential oil components in *O. vulgare* subsp. *vulgare* and *O. vulgare* subsp.*gracile* under salinity stress

No	Components	RI	*O. vulgare* subsp. *gracile*				*O. vulgare* subsp. *vulgare*			
			Control	25 mM	50 mM	100 mM	Control	25 mM	50 mM	100 mM
1	*α*-Thujene	923	1.56 ± 0.00	1.55 ± 0.01	1.29 ± 0.01	1.16 ± 0.01	1.66 ± 0.00	1.69 ± 0.05	1.24 ± 0.02	1.17 ± 0.00
2	*α*-Pinene	930	0.60 ± 0.01	0.64 ± 0.01	0.56 ± 0.01	0.53 ± 0.01	0.63 ± 0.01	0.66 ± 0.01	0.47 ± 0.01	0.48 ± 0.01
3	Sabinene	970	-	-	-	-	0.23 ± 0.00	0.24 ± 0.00	-	-
4	1-Octanone-3-ol	973	0.96 ± 0.00	0.84 ± 0.01	0.79 ± 0.01	0.83 ± 0.01	1.25 ± 0.01	1.17 ± 0.01	0.9 ± 0.05	0.95 ± 0.01
5	3-Octanone	981	1.27 ± 0.03	1.22 ± 0.01	1.27 ± 0.01	1.72 ± 0.01	0.77 ± 0.00	0.62 ± 0.01	0.59 ± 0.00	0.6 ± 0.01
6	*β*-Myrcene	986	1.66 ± 0.03	1.74 ± 0.00	1.56 ± 0.01	1.65 ± 0.00	1.64 ± 0.01	1.67 ± 0.01	1.3 ± 0.1	1.36 ± 0.03
7	*α*-Phellandrene	1003	-	0.3 ± 0.1	-	-	0.32 ± 0.01	0.27 ± 0.01	-	0.24 ± 0.00
8	*α* -Terpinene	1014	2.5 ± 0.1	2.46 ± 0.02	2.17 ± 0.01	2.18 ± 0.00	2.95 ± 0.02	2.6 ± 0.01	2.37 ± 0.01	2.48 ± 0.00
**9**	***p*-Cymene**	**1022**	**5.14 ± 0.05**	**5.4 ± 0.4**	**6.93 ± 0.2**	**11.81 ± 0.2**	**6.13 ± 0.05**	**8.45 ± 0.5**	**8.72 ± 0.5**	**8.61 ± 0.05**
10	*β*-Phellandrene	1027	0.51 ± 0.01	0.54 ± 0.00	0.47 ± 0.01	0.53 ± 0.01	0.62 ± 0.00	0.62 ± 0.01	0.51 ± 0.01	0.54 ± 0.00
11	1,8-Cineole	1029	-	-	-	0.35 ± 0.01	0.36 ± 0.00	0.42 ± 0.00	0.43 ± 0.00	0.47 ± 0.01
12	*β*-Ocimene	1033	1.41 ± 0.01	1.79 ± 0.02	1.74 ± 0.02	1.46 ± 0.01	1.07 ± 0.01	0.9 ± 0.05	0.63 ± 0.01	0.9 ± 0.05
**13**	***γ-*Terpinene**	**1057**	**12.33 ± 0.05**	**12.36 ± 0.5**	**10.55 ± 0.05**	**10.53 ± 0.2**	**14.73 ± 0.05**	**15.64 ± 0.05**	**13.99 ± 0.05**	**13.52 ± 0.05**
14	*cis*- Sabinene hydrate	1065	0.88 ± 0.01	0.65 ± 0.01	0.65 ± 0.00	0.98 ± 0.01	0.88 ± 0.00	0.62 ± 0.01	0.69 ± 0.01	0.75 ± 0.01
15	*β*-Pinene	1097	0.31 ± 0.00	0.29 ± 0.00	-	0.31 ± 0.00	0.7 ± 0.05	0.59 ± 0.00	-	0.52 ± 0.00
16	Borneol	1166	-	-	-	-	-	0.39 ± 0.00	0.28 ± 0.00	0.34 ± 0.00
17	Terpinene-4-ol	1177	0.54 ± 0.02	0.68 ± 0.01	0.66 ± 0.02	0.51 ± 0.01	0.61 ± 0.01	0.76 ± 0.01	0.7 ± 0.1	0.72 ± 0.01
18	*α*-Terpineol	1190	0.56 ± 0.03	0.56 ± 0.01	0.52 ± 0.01	0.45 ± 0.01	0.37 ± 0.01	1.12 ± 0.01	0.92 ± 0.01	0.93 ± 0.01
**19**	**Carvacrol methyl ether**	**1241**	**3.43 ± 0.01**	**5.57 ± 0.01**	**4.25 ± 0.01**	**4.04 ± 0.00**	**9.31 ± 0.01**	**8.95 ± 0.01**	**9.35 ± 0.01**	**8.4 ± 0.1**
**20**	**Thymol**	**1288**	**2.76 ± 0.2**	**1.73 ± 0.2**	**2.73 ± 0.2**	**2.32 ± 0.2**	**6.66 ± 0.2**	**4.62 ± 2**	**3.97 ± 2**	**5.66 ± 0.3**
**21**	**Carvacrol**	**1304**	**60 ± 1.15**	**57.6 ± 1.18**	**59.6 ± 1.09**	**56.3 ± 1.09**	**40.93 ± 1.14**	**42.08 ± 0.99**	**47.36 ± 1.15**	**45.2 ± 1.09**
22	trans-Caryophyllene	1420	0.75 ± 0.01	0.67 ± 0.01	0.75 ± 0.02	0.52 ± 0.01	1.23 ± 0.01	1.01 ± 0.00	1.09 ± 0.00	1.33 ± 0.01
23	*β*-Bisabolene	1506	-	0.3 ± 0.05	0.31 ± 0.00	-	0.41 ± 0.00	0.35 ± 0.00	0.3 ± 0.05	0.4 ± 0.05
24	δ-Cadinene	1522	-	0.3 ± 0.05	0.3 ± 0.05	-	0.41 ± 0.00	0.36 ± 0.00	0.28 ± 0.00	0.41 ± 0.00
25	*cis*-α-Bisabolene	1539	2.74 ± 0.00	2.7 ± 0.05	2.82 ± 0.01	1.62 ± 0.01	3.56 ± 0.01	3.19 ± 0.01	3.11 ± 0.01	3.62 ± 0.01
26	(-)-Spathulenol	1579	-	-	-	-	0.21 ± 0.01	-	-	-
27	Caryophyllene oxide	1586	-	-	-	-	0.23 ± 0.01	-	-	-
Class and subclass of compounds										
Monoterpenes			93.65	93.18	93.02	94.6	89.19	91.53	92.23	91.57
Monoterpene hydrocarbons			27.46	28.28	26.44	31.59	31.93	35.07	30.84	31.5
Oxygenated monoterpenes			66.19	64.9	66.58	63.01	57.26	56.46	61.39	60.07
Sesquiterpenes			3.49	3.97	4.18	2.14	6.05	4.91	4.78	5.76
Sesquiterpene hydrocarbons			3.49	3.97	4.18	2.14	5.61	4.91	4.78	5.76
Oxygenated sesquiterpenes			-	**-**	**-**	-	0.44	-	-	-
Others			2.77	2.74	2.72	3.06	2.63	2.55	2.19	2.27
Total identified			99.91	99.89	99.92	99.8	97.87	98.99	99.2	99.6

*RI *Retention indices, Data are mean ± SE (*n* = 3)

According to the results, monoterpenes were the main groups of the identified components in both subspecies. The essential oil of subsp. *gracile* contained monoterpenes (93.65%, 93.18%, 93.02% and 94.6%) at different levels of salinity, respectively. Oxygenated monoterpenes had the highest percentage in the subclass of monoterpenes. Of these, carvacrol, carvacrol methyl ether and thymol were the major components. 1,8-cineole, as an oxygenated monoterpene, was detected only at 100 mM NaCl in subsp. *gracile*. Monoterpene hydrocarbons are the second subclass of the monoterpenes, among which γ-terpinene and *ρ*-cymene were identified as the dominant components. Sesquiterpene hydrocarbons were the next subclass of compounds found in subsp. *gracile* oil that reached the highest percentage (4.18%) at 50 mM NaCl, and cis-α- bisabolene was identified as the major component. In addition, oxygenated sesquiterpenes were not detected in subsp*. gracile*. In contrast, the oil of subsp. *vulgare* contained monoterpenes (89.19%, 91.53%, 92.23% and 91.57%) and sesquiterpenes (6.05%, 4.91%, 4.78% and 5.76%) at different levels of salinity, respectively. Oxygenated monoterpenes were the most dominant subclass of compounds in subsp. *vulgare*. Of these, carvacrol, carvacrol methyl ether and thymol were the major components. Furthermore, the highest percentage (61.39%) of oxygenated monoterpenes was found at 50 mM NaCl. The highest monoterpene hydrocarbons (35.07%) as the second subclass of compounds were observed at 25 mM NaCl, of which γ-terpinene and *ρ*-cymene were identified as predominant components. Sesquiterpene hydrocarbons were another dominant subclass of compounds with cis-α- bisabolene as the major component. Spathulenol and caryophyllene oxide are the only oxygenated sesquiterpenes identified at non-stress treatments in subsp*. vulgare*. Moreover, α-phellandrene was detected at 25 mM NaCl in subsp*. gracile*. Whereas, in subsp*. vulgare* it was not detected only at 50 mM NaCl. Sabinene, as a monoterpene hydrocarbon, was not identified in *gracile* subspecies but was found at non-stress treatment and 25 mM NaCl in *vulgare* subspecies (Table 1).

Correlation analysis showed a negative relationship between γ-terpinene and *p*-cymene in subsp. *vulgare*. Also in this subspecies, a negative relationship was obtained between γ-terpinene and thymol with carvacrol, whereas the correlation between *p*-cymene and carvacrol was positive. In addition, the correlation between γ-terpinene and *p*-cymene with thymol was negative (Fig. 6a). In contrast in subsp. *gracile*, a negative correlation was observed between γ-terpinene and *p*-cymene. Also, a negative relationship was obtained between *p*-cymene and carvacrol, as well as γ-terpinene with thymol. Furthermore, a positive relationship was observed between carvacrol with thymol and γ-terpinene with carvacrol (Fig. 6b).

### Gene expression levels

**Fig. 5:**
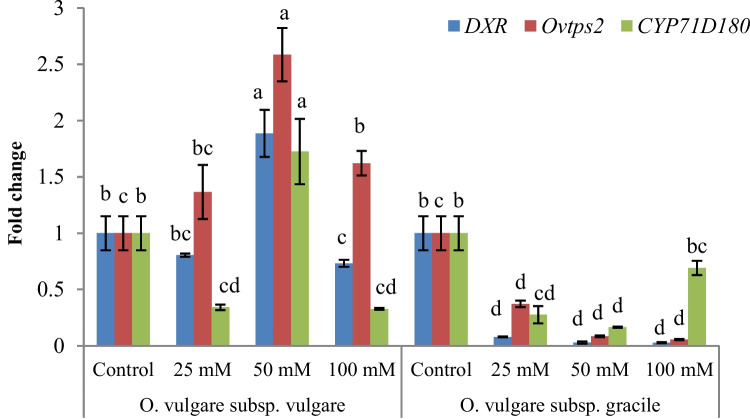
Relative expression (RE) of *DXR*, *Ovtps2*, *CYP71D180* genes in *Origanum vulgare* (subsp. *vulgare* and subsp. *gracile*) under salinity stress. Error bars indicate standard error of the mean (*n* = 3). Columns with different letters have significant differences (*p* < 0.05)

To partly unravel the molecular mechanism by which salinity stress alters the content of essential oil in two studied oregano subspecies, the expression levels of *DXR*, *Ovtps2* and *CYP71D180* genes were investigated under various salinity levels in these subspecies for the first time. The expression levels of studied genes were significantly affected by salinity treatments, subspecies and their interaction. The highest *DXR* expression was observed at 50 mM NaCl in *vulgare* subspecies, while the lowest expression of that was obtained at *gracile* subspecies under salinity stress. Furthermore, the highest expression of *Ovtps2* was observed at 50 mM NaCl in *vulgare* subspecies. However, in *gracile* subspecies, the relative expression of this gene decreased with increasing salinity. Similar to the *DXR* gene, the highest relative expression of *CYP71D180* was obtained at 50 mM salinity in *vulgare* subspecies. Whereas, in *gracile* subspecies, the expression of this gene decreased with increasing salinity up to 50 mM, then increased at 100 mM salinity (Fig. 5).

**Fig. 6:**
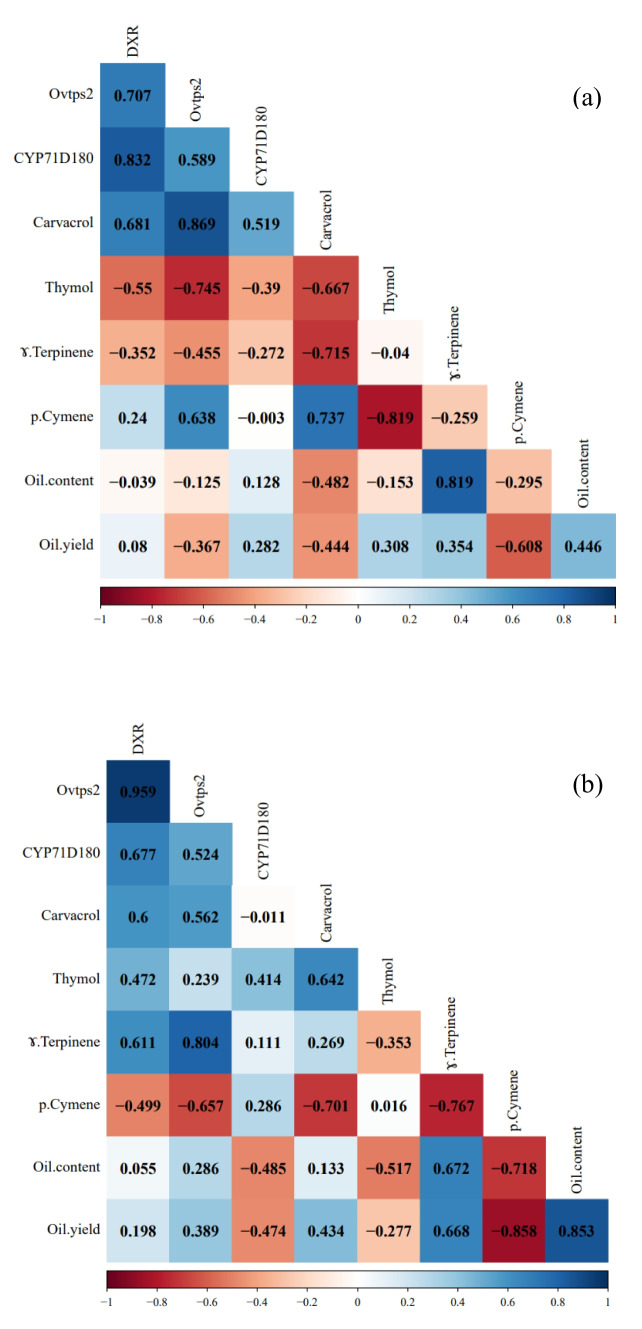
Pearson’s correlation coefficients between the studied genes, essential oil content and yield and essential oil components in *O. vulgare* subsp. *vulgare* (**a**) and *O. vulgare* subsp. *gracile* (**b**). Positive and negative correlations are displayed in blue and red, respectively

A positive relationship was observed between the expression of *DXR*, *Ovtps2* and *CYP71D180* genes with carvacrol in *vulgare* subspecies, while, the correlation of these genes with thymol content was negative. Also, a negative correlation was found between the relative expression of *Ovtps2* gene and γ-terpinene, while the correlation of this gene with *p*-cymene was positive in this subspecies (Fig. 6a). In contrast, in *gracile* subspecies, a positive correlation was obtained between the relative expression of *Ovtps2* and γ-terpinene, whereas the correlation of this gene with *p*-cymene was negative. There was a negative relationship between the expression of *CYP71D180* and carvacrol, while a positive correlation was obtained between *DXR* and *Ovtps2* genes expression with carvacrol content. Also, in *gracile* subspecies, the correlation between the three studied genes and thymol content was positive (Fig. 6b).

## Discussion

To deal with salinity, plants adjust their growth and development behaviors along with an organizing between primary and secondary metabolites [48]. The results of the several investigations demonstrate that the biosynthesis of secondary metabolites in medicinal plants is seriously affected by environmental factors [23, 49–51]. Furthermore, the difference between the content and composition of essential oil in medicinal plants depends on the various factors such as cultivar, genetics and environmental conditions [52]. However, studies have shown that these changes may be caused through the different expression of the enzymes involved in the production of these compounds under salinity conditions [53, 54]. In this investigation, essential oil content influenced by salinity stress and subspecies. The highest percentage of essential oil was achieved for subsp. *gracile* at 25 mM salinity. However, the essential oil content decreased at 50 and 100 mM NaCl stress. Under moderate salinity stress, the stimulation of essential oil production can be due to the higher density of essential oil glands [55]. Moreover, the increment of essential oil contents in plants may be due to the reduction of primary metabolites by salinity and the improvement of intermediary products availability for secondary metabolites synthesis [54, 55]. According to the previous studies, the essential oil content increased with the intensity of salinity in *Salvia officinalis* [56] and *Ocimum basilicum* [55]. However, the essential oil content decreased by increasing salinity in *O. majorana* [57] and *Mentha piperita* [58]. Moreover, the highest essential oil yield was observed at low salinity level in subsp. *gracile*. The essential oil yield in subsp. *vulgare* decreased with increasing salinity. Similarly, high salinity levels led to a decline in essential oil yield in some plant species such as, *Trachyspermum ammi* [59] and *Matricaria* sp. [60].

The chemical composition of *O. vulgare* essential oil has been studied in several researches [9, 15, 25, 61]. There is a high variety in essential oil composition of this plant. The main composition of essential oil in *O. vulgare* is thymol, carvacrol, γ-terpinene, *p*-cymene, β-myrcene and β-bisabolene [2, 9, 13]. In this study, the main components under salinity treatments and in both subspecies were carvacrol, γ-terpinene, *p*-cymene, thymol, carvacrol methyl ether and cis-*α*-bisabolene. It can be considered that the accumulation of some main compounds as a defense mechanism in medicinal plants by inducing changes in cellular metabolism adapts them to stress conditions [62]. Salinity stress can affect the essential oil composition of plants depending on its severity. In previous reports, percentage of main compounds enhanced with severity of NaCl stress in comparison with non-stress conditions, in *Salvia officinalis* [48, 53, 63], *S. mirzayanii* [64] and *Ocimum basilicum* [55].

Monoterpenes in plants have a high commercial value industrially and can be used in the perfume, anti-cancer and pesticide industries [65]. Two dominant components of oregano essential oil are phenolic monoterpenes, thymol and carvacrol, which are well known their anti-vegetarian, antimicrobial, medicinal and antioxidant activities [25]. In the present research, an early gene (*DXR*), a middle gene (*Ovtps2*) and a last gene (*CYP71D180*) in MEP pathway, involved in thymol and carvacrol biosynthesis [30, 31] were evaluated, which showed that salinity stress significantly affected their expression. This might be due to the role of terpenes in defense pathways and signal transduction in oregano. Based on the results, a positive correlation was obtained between the expression of *DXR* with *Ovtps2* and *CYP71D180* in both subspecies. According to the previous studies, γ-terpinene and* p*-cymene are the main precursors of thymol and carvacrol in oregano and thyme, which are synthesized by the γ-terpinene synthase enzyme from geranyl diphosphate [25, 29]. The results of *Ovtps2* gene expression in subsp. *vulgare* indicated that salt stress increased the expression of this gene compared with control. Furthermore, *Ovtps2* as an intermediate gene in the pathway of thymol and carvacrol biosynthesis was more affected than *DXR* and *CYP71D180* genes at all salinity levels. In oregano, the contents of thymol and carvacrol in leaves are related to the expression of *Ovtps2* [25]. In this study, the relative expression of *Ovtps2* was increased in subsp. *vulgare* at 50 mM salinity, while the percentage of γ-terpinene (as a precursor of thymol and carvacrol) decreased. In other words, a negative correlation was found between the expression of *Ovtps2* and γ-terpinene at this salinity level. The lack of congruence between the transcriptional levels of the genes and their corresponded compounds (less gene expression but more compound production) may be due to the effect of stress on the enzymatic activity or some changes in transcription and post-translational processes [50, 66]. Post-translational modifications of proteins are very important factor in regulating the plant response to the stress conditions [67] and can regulate protein function, location, half-life and protein interactions to reduce the potential damage caused by environmental stresses [68]. However, the activity of the enzymes under salinity stress have not been studied in this investigation.

Also, the highest expression of the studied genes and carvacrol content was observed in *vulgare* subspecies at 50 mM salt stress. The gene expression levels may be variable depending on the stress and plant species [69]. The higher expression of these genes in retort to moderate salinity stress may reverberate the elevation of phenolic monoterpenes such as carvacrol. Similarly, the higher expression of biosynthesis genes in response to abiotic elicitors has been associated with the increment of the corresponding metabolites in plants such as *Tanacetum parthenium* (L.) Sch. Bip. [70] and *Nigella sativa* L. [71]. On the contrary, despite the higher expression of *DXR*, *Ovtps2* and *CYP71D180* genes at 50 mM salinity, the content of thymol decreased. In previous studies, high transcription levels and high carvacrol production in thyme and oregano were correlated with genes encoding *CYP71D180* and *CYP71D181* [72]. Therefore, the reduction of thymol can be attributed to *CYP71D*. In the present study, severe salinity stress reduced the expression of *CYP71D180* in subsp. *vulgare*, which was consistent with the trend of carvacrol changes. It can be concluded that salinity stress probably reduces the amount of carvacrol in *vulgare* subspecies through reducing the expression of *CYP71D180*. However, in plants treated with sever salinity concentrations, despite a decline in the expression of *CYP71D180*, the biosynthesis of thymol (as a carvacrol isomer) increased, indicating that, other *CYP450* homologues are likely involved in increasing thymol production. Noteworthy, 11 sequences of *CYP450* gene have been isolated from oregano and thyme by Crocoll et al. [25]. Previous studies have shown that, there is a significant relation between the activity of *CYP450* family enzymes and the production of monoterpenes such as carvacrol and thymol in oregano [30, 31]. In the formation of thymol and carvacrol from *γ*-terpinene, the aromatic hydrocarbon *p*-cymene has been proposed as an intermediary [73], however, its participation and the nature of the enzymes involved in the formation of the aromatic ring are still unknown [72]. In this study, the trend of *Ovtps2* changes was consistent with *p*-cymene at different salinity levels.

Also, a positive relationship was observed between the relative expressions of the studied genes with carvacrol in subsp. *vulgare* and inversely, a negative relationship was obtained with thymol production. In addition, a negative relationship was observed between γ-terpinene and carvacrol in *vulgare* subspecies. Similarly, Morshedloo et al. [31] stated a negative correlation between γ-terpinene and carvacrol in *O. vulgare* subsp. *gracile* under drought stress. Also, a negative relationship between carvacrol and thymol in this subspecies was found. Hence, it can be concluded that γ-terpinene is a precursor for carvacrol. On the other hand, carvacrol is an isomer of thymol and they can be converted to each other. In *gracile* subspecies, a positive correlation was observed between the relative expression of *DXR* and *Ovtps2* with carvacrol, but the correlation between *CYP71D180* and carvacrol was negative. Presumably, the negative association between carvacrol and *CYP71D180* may be due to the role of other enzymes of cytochrome family in this pathway [31]. It has been demonstrated that *Ovtps2,* as the main terpene synthase, produces the half of the total terpenes content [30]. In this study, a direct relationship was obtained between the expression of *Ovtps2* and thymol synthesis in subsp. *gracile*. The findings of this investigation are in line with Crocoll et al. [30] who reported that there is a positive correlation between *Ovtps2* gene expression and γ-terpinene and thymol production in *O*. *vulgare*.

## Conclusions

In overall, the result revealed that the essential oil content increased up to 25 mM NaCl and then decreased. Also, *gracile* subspecies had a higher essential oil content than *vulgare* subspecies. No significant difference was found between NaCl treatments (0, 25 and 50 mM) in terms of essential oil yield in both subspecies. Carvacrol, as the main component of essential oil, decreased with increasing salinity levels in subsp. *gracile* but increased in subsp. *vulgare*. The highest expression of *DXR*, *Ovtps2* and *CYP71D180* genes was observed at 50 mM NaCl in subsp. *vulgare*. A positive relationship was observed between the expression of *DXR*, *Ovtps2* and *CYP71D180* with carvacrol content in subsp. *vulgare* and between the expression of *DXR* and *Ovtps2* with carvacrol content in subsp. *gracile*. While, a negative association was observed between the expression of *DXR*, *Ovtps2* and *CYP71D180* with thymol content in subsp. *vulgare*. In contrast, the correlation of *DXR*, *Ovtps2* and *CYP71D180* with thymol content in subsp. *gracile* was positive. Therefore, due to the pharmacological properties of carvacrol and its economic value in the food and cosmetics industries, it can be suggested to enhance its production by increasing the expression of *DXR*, *Ovtps2* and *CYP71D180* genes under controlled conditions in the future studies. Also, study the expression of salt-inducible genes/transporters in both subspecies and their relationship with genes involved in MEP pathway under salinity conditions, as well a transcriptome analysis using RNA-seq in both subspecies might lead to a comprehensive view regarding the MEP pathway in the studied subspecies and genetically closed genera.

## Materials and methods

### Plant material and growing conditions

Seeds of two subspecies of Oregano (*O. vulgare* subsp. *vulgare* and *O. vulgare* subsp. *gracile*) were obtained from the collection of medicinal plants in Department of Horticultural Science, Urmia University (West Azerbaijan province, Iran). The plant samples were identified by Hossien Maroofi (Research Center of Agriculture and Natural Resources of Kurdistan, Sanandaj, Iran). Voucher specimens were deposited at the herbarium in Department of Horticultural Science, Faculty of Agriculture, Urmia University, Iran. The experiment was performed as a factorial in a completely randomized design (CRD) with three replications during 2019–2020. Seeds of two subspecies were planted in plastic pots in research greenhouse of Urmia University. Each pot (diameter: 25 cm and height: 30 cm) was filled with a 3:2 ratio of soil and sand. The physical and chemical characteristics of the soil used in the pots were: pH (8.02), EC (1.27 ds m^−1^), organic material (0.62%), total nitrogen (0.12%), available P (9.45 ppm), exchangeable K (0.46 meq/100g soil), and texture (sandy loam). The greenhouse temperature was in the range of 20 ± 2 to 28 ± 2°C with 50–60% relative humidity under natural sunlight. After seed germination, the seedlings were thinned and finally 7 plants kept in each pot. The plants were irrigated evenly with ordinary water until reaching the stage of 6–8 leaves. After this stage, they were subjected to salinity stress for 45 days (until the flowering stage). The salinity treatments applied, included four levels of saline irrigation (0, 25, 50 and 100 mM NaCl). To avoid sudden shock from salinity stress, salinity treatments gradually reached the final concentration during the three irrigation stages. At the full flowering stage, 10 fully developed leaves were harvested from each treatment and transferred to a -70 °C freezer to evaluate the relative expression of *DXR*, *Ovtps2* and *CYP71D180* genes. Then, the aerial parts of plants were cut from 10 cm above the soil in order to essential oil extraction and analysis.

### Essential oil extraction

The aerial parts of oregano were shade dried, and then plant material (20 g) was subjected to hydro-distillation (Clevenger apparatus, 2.5 h) for essential oil extraction. The essential oil content was expressed as volume per dry weight percentage (%v/w). The collected essential oils were dehydrated over anhydrous sodium sulfate and stored in dark sealed vials at low temperature (4°C) till analysis.

### GC–MS analysis of plant volatiles

Gas chromatography/mass spectrometry (GC–MS) was used for analysis of essential oil components. An Agilent 7890 gas chromatograph paired with a 5975A mass spectrometer equipped with a HP-5 MS capillary column (5% Phenyl Methylpolysiloxane, 30 m length, 0.25 mm i.d., 0.25 μm film thickness) (Agilent Technologies, Wilmington-DE, USA), was used for GC–MS analysis. The oven temperature program was adjusted for 3 min (at 80°C), then raised at 10°C min^−1^ to 200°C, kept for 15 min at 200°C. The temperatures applied to the injector, transfer line and ion resource were 240°C, 280°C and 230°C, respectively. The carrier gas used (with a flow rate of 1 mLmin^−1^ and an electron impact (EI) of 70 eV) was helium. The injector was set in a split mode (split ratio of 1:50) and injection volume was 1.0 μL. Mass spectra were scanned in the range of 40–500 amu. The constituents of essential oil were determined by using the calculated linear retention indices (Wiley 2007; NIST 2005) and mass spectra with those reported in the NIST 05 and Wily 07.

### RNA isolation and cDNA synthesis

Total RNA of *O. vulgare* leaves was extracted using RNX plus™ kit according to the manufacturer's instructions (Sinaclon, Iran). After evaluating the quality and quantity of RNA using 1% agarose gel electrophoresis and nanodrop ND-1000, cDNA was synthesized using Revert Aid™ First Strand cDNA Synthesis Kit (Thermo Fisher Scientific, USA) according to the instructions of the manufacturer (Thermo Scientific, USA). Negative control reactions using reverse transcriptase minus (-RT) and non-template control (NTC), was performed to ensure no genomic DNA contamination and for reagent contamination, respectively.

### Real time PCR reactions

The relative expression of the genes was investigated using Real time PCR (Rotor gene Q-Pure Detection-Qiagen) in the treated plants compared with the control. Gene specific primer pairs were selected from previous studies [25, 31]. Real time PCR reactions were carried out by considering three biological replications in the final volume of 12.5 μL using Maxima ® SYBR-Green/ROX qPCR Master mix kit (Thermo Fisher Scientific, USA), according to the manufacturer's instructions. Initial activation of the enzyme was done at 95°C for 10 min in one cycle, followed by 40 cycles including denaturation at 95°C for 10 s, annealing at 58–60°C for 15 s and fluorescence data collection at 72°C for 20 s. The actin gene was used as the reference gene to normalize the data. The relative expression of the studied genes was calculated after obtaining Ct by ΔΔCt method [74].

### Statistical analysis

The experiment was performed as a factorial experiment in CRD with three replications. Data obtained were subjected to analysis of variance (ANOVA) followed a comparison of the means using Duncan’s multiple range test at *p* < 0.05 level using SAS 9.2 software. The relevance between the main constituents of essential oil and gene expression level were estimated using the Pearson’s correlation coefficient by R software.

## Data Availability

The datasets used and/or analyzed during the current study available from the corresponding author on reasonable request.
